# Development of ordered mesoporous carbon-based electrodes for the electrocatalytic oxidation of furfural

**DOI:** 10.3389/fchem.2026.1745807

**Published:** 2026-05-25

**Authors:** Paolo Squillaci, Donatella Chillè, Georgia Papanikolaou, Siglinda Perathoner, Gabriele Centi, Paola Lanzafame

**Affiliations:** Department ChiBioFarAm - Industrial Chemistry, University of Messina, INSTM CASPE (Laboratory of Catalysis for Sustainable Production and Energy) and ERIC aisbl, Messina, Italy

**Keywords:** 5-HFN, electrodes, furfural electro-oxidation, ordered mesoporous carbons, SBA-15 templated carbon

## Abstract

The electrocatalytic valorization of furfural, a key biomass-derived platform molecule, offers a sustainable pathway for the synthesis of value-added chemicals under mild conditions. In this study, we report the development and application of nanostructured carbon-based electrodes, synthesized via a hard templating approach using SBA-15 silica and carbon precursors (furfuryl alcohol, FA, and glycerol, GLY), for the electro-oxidation of furfural. The resulting ordered mesoporous carbons (OMC_FA_ and OMC_GLY_), along with a gold nanoparticle-decorated variant (Au@OMC_GLY_), were thoroughly characterized by XRD, N_2_ physisorption, SEM-EDX, and Raman spectroscopy to elucidate their structural, textural, and electrochemical properties. Electrochemical performance was evaluated in a microflow cell under acidic and alkaline conditions, with 0.25 M HClO_4_ as the optimized electrolyte for selective conversion. Among the tested electrodes, OMC_GLY_ exhibits the highest Faradaic efficiency (83%) towards 5-hydroxy-2(5H)-furanone (5-HFN), outperforming both the benchmark reduced graphene oxide and the Au-modified OMC_GLY_, the latter favoring maleic acid formation at higher potentials. Structure-activity relationships suggest that enhanced surface area, pore structure, and graphitic character of OMC_GLY_ synergistically contribute to its superior catalytic behavior. These findings demonstrate the potential of tailored mesoporous carbon architectures as efficient and sustainable electrocatalysts for biomass upgrading.

## Graphical Abstract

**Figure:**
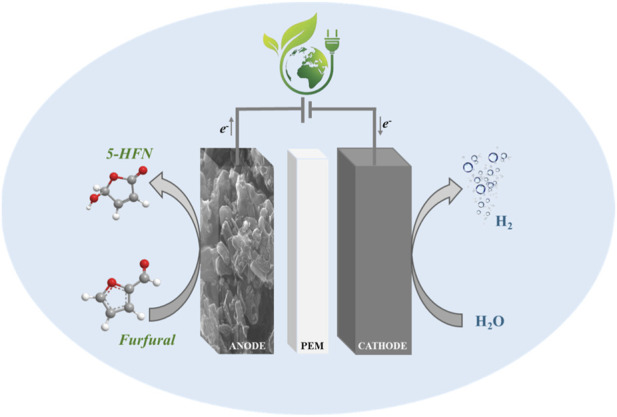

## 1 Introduction

The growing depletion of fossil resources and associated environmental issues have increased international efforts to use renewable feedstocks to produce high-value chemicals in an environmentally friendly and sustainable manner (Mika et al., 2018; Tian et al., 2024; Sudarsanam et al., 2018; Papanikolaou et al., 2022a). Over the last few decades, efficient and environmentally benign energy conversion technologies have driven extensive research into advanced materials and electrochemical processes (Xia et al., 2022; Amir et al., 2023; Ganiyu and Martinez-Huitle, 2020). The electrocatalytic oxidation of biomass-derived compounds is thus becoming a promising route for clean energy generation and value-added chemical synthesis (Akhade et al., 2020; Liu and Li, 2021).

Furfural is considered one of the key bio-based platform molecules derived from hemicellulosic biomass and constitutes an attractive renewable substrate due to its abundance and versatile reactivity. Furfural can be transformed into a series of value-added products, such as furfuryl alcohol and 2-methylfuran, by catalytic hydrogenation. In contrast, its electrocatalytic oxidation leads to the formation of relevant derivatives such as furoic acid, 5-hydroxyfuran-2(5H)-one (5-HFN), and maleic acid (Sabri et al., 2022; Bharath and Banat, 2021). Among them, the 5-HFN compound is of particular value as an intermediate with significant biological activity, and it plays a fundamental role in the synthesis of bioactive molecules and fine chemicals (Kumar and Pandey, 2000).

Different thermocatalytic and photocatalytic routes have been reported so far for 5-HFN production from furfural or its derivatives, such as furfuryl alcohol and furoic acid (Rodenas et al., 2018; Thiyagarajan et al., 2020; Kar et al., 2022; Kubota and Choi, 2018). However, both approaches face intrinsic limitations. The selective formation of 5-HFN remains challenging, as it readily undergoes further oxidation to maleic acid. Moreover, photocatalytic processes often rely on photosensitizers that are difficult to recycle and environmentally detrimental, limiting their practical applicability (Rodenas et al., 2018; Fan et al., 2022). In contrast, electrocatalytic oxidation represents a sustainable alternative that operates under ambient conditions and eliminates the need for toxic chemical oxidants (Sohail and Wattanakit, 2025; Chen et al., 2022).

Wu et al. (2019) recently identified metal chalcogenides as efficient electrocatalysts for the conversion of furfural to 2-hydroxyfuranone (HFO) in an ([Et_3_NH]NO_3_-MeCN-H_2_O) electrolyte system. Among these, copper sulfide (CuS) nanosheets exhibited the highest performance, achieving a 5-HFN yield of 58.7% along with excellent long-term stability. In another study, a Mn-Ce-based mixed oxide (MnCeO_x_), derived from the pyrolysis of a Mn-Ce-BTC metal-organic framework, demonstrated remarkable activity and selectivity for furfural oxidation, producing MA with yields up to 67.4% and a Faradaic efficiency of 65.9% at 1.7 V vs. Ag/AgCl (Yuan et al., 2024). The superior performance of MnCeO_x_ compared to Mn_3_O_4_ and CeO_2_ was attributed to the high density of surface oxygen vacancies, though challenges related to scalability and product separation remain.

Additionally, furfural electro-oxidation to furoic acid has been demonstrated on both Pt/C and Au electrodes in acidic electrolytes (Román et al., 2019; Román et al., 2020). On Pt/C, the reaction exhibits low activity and moderate selectivity below 1.0 V vs. RHE. In comparison, Au electrodes display significantly higher activity (∼2 μA⋅cm_-2_ Au at 1.0 V vs. RHE) and up to 96% ± 6% Faradaic efficiency toward furoic acid at 0.8 V vs. RHE. Through spectroscopic and computational analysis, surface-bound furoate intermediates were identified as essential species, with product desorption as the rate-limiting step; molecular self-assembly characteristics influence selectivity.

Despite such advances, the high cost, scarcity, and limited durability of metal-based electrocatalysts under acidic conditions remain significant barriers to their large-scale deployment. Hence, increasing effort has been devoted to the development of efficient, metal-free or metal-supported carbon-based materials as sustainable alternatives for biomass valorisation (Wang et al., 2025; Zhu et al., 2024). Among these, ordered mesoporous carbons (OMCs), typically synthesized via nanocasting using SBA-15 silica templates, stand out for their high surface area, uniform mesopore distribution, tunable structural properties, and excellent electrical conductivity (Parmentier et al., 2004; Ignat et al., 2010; Pérez-Rodríguez et al., 2019). Moreover, the ability to modulate their micro- and mesoporosity and to introduce specific surface functionalities or metal nanoparticles enables fine-tuning of their catalytic properties and surface chemistry (Janus et al., 2020; Liu et al., 2017; Bai et al., 2010). The combination of ordered porosity, high electrical conductivity, and accessible active sites promotes rapid charge and mass transport, facilitating efficient electro-oxidation reactions (Zhang et al., 2020; Xue et al., 2020; Xu et al., 2022).

In this work, OMCs synthesized from furfuryl alcohol (FA) and glycerol (GLY) were investigated as efficient electrocatalysts for the oxidation of furfural under mild conditions. The study aims to shed light on how textural order, pore accessibility, and electronic conductivity govern the reaction mechanism and product selectivity. To this end, a systematic comparison between the two OMCs was designed to show how precursor choice controls graphitic character, defect density, and charge-transfer capability, parameters that ultimately determine catalytic efficiency. In addition, the influence of the electrolyte environment was evaluated to establish structure-electrolyte-selectivity correlations, particularly in directing the formation of 5-HFN versus furoic acid. To further probe surface reactivity, gold nanoparticles were incorporated onto the most conductive OMC framework. Rather than serving as a mere activity enhancer, Au acts as a diagnostic probe to explore the effects of localized electronic enrichment and metal-support interactions on the oxidation pathway (Kim et al., 2024).

Overall, the goal of this study is to provide a comprehensive understanding of how the structural and electronic features of mesostructured carbons dictate the electrocatalytic oxidation of furfural. By correlating physicochemical characteristics with electrochemical performance, this work highlights the potential of OMCs as sustainable, metal-free platforms for the selective electro-oxidation of biomass-derived compounds. It identifies key parameters for their rational design and optimization in future continuous-flow electrochemical systems.

## 2 Materials and methods

### 2.1 Catalysts synthesis and characterization

#### 2.1.1 Ordered mesoporous carbon materials synthesis

Ordered mesoporous carbon materials, denoted as OMC_FA_ and OMC_GLY_, were synthesized using a hard-templating approach with mesoporous silica SBA-15 as the template, which was prepared in the laboratory following procedures reported in the literature (Ignat et al., 2010; Janus et al., 2020; Abate et al., 2011). Two different carbon precursors were employed: FA and GLY, respectively. For both syntheses, the SBA-15 template was impregnated under acidic conditions. In the case of OMC_FA_, a hydrochloric acid solution was used as the catalyst, whereas for OMC_GLY_, sulfuric acid was employed. The mixtures were heated at 100 °C for 6 h to promote polymerization of the carbon precursors within the silica framework. The resulting solids were filtered, dried, and then pyrolyzed under a continuous N_2_ flow in a quartz tubular reactor.

The carbonization of the FA-derived composite was performed at 850 °C (heating rate: 5 °C·min^-1^) for 4 h, while the GLY-derived material was pyrolyzed at 800 °C (heating rate: 1 °C·min^-1^) for 5 h. After cooling to room temperature, the silica template was removed by chemical etching in aqueous HF, under stirring overnight at ambient temperature. The resulting carbon materials were thoroughly washed with distilled water and ethanol, filtered, and finally dried in an oven at 40 °C overnight to yield the ordered mesoporous carbons OMC_FA_ and OMC_GLY_.

#### 2.1.2 Deposition of gold nanoparticles on OMC

Gold nanoparticles were deposited onto OMC_GLY_ using the sol-immobilization method (AuNPs). The carbon material was suspended in an aqueous solution containing different aliquots of HAuCl_4_·3H_2_O and polyvinyl alcohol (PVA) to achieve an Au/PVA weight ratio equal to 1:1. Afterwards, a solution of NaBH_4_ was added drop by drop to the suspension and the mixture was maintained under continuous stirring for 3 h at room temperature (Papanikolaou et al., 2021; Villa et al., 2013). The as-prepared gold-supported carbon replica Au@OMC_GLY_ was washed until the pH reached 7, filtered, dried at 80 °C for 12 h, and finally calcined in air at 200 °C for 2 h (2 °C⋅min^-1^). The final theoretical Au-metal loading was 5 %wt.

### 2.2 Morphological and textural characterization

XRD was used to characterize the structure of SBA-15 and its carbon replicas. As regards the SBA-15, data were recorded by means of two distinct diffractometers: a D2 Phaser diffractometer Bruker equipped with a Cu tube (λ = 1.54056 Å) and operating at high angle (2θ range = 10°–90°) with an angular step size of 0.025°·s^-1^; a Bruker D8 Advance diffractometer acting at low angle (2θ range = 0.6°–3°) with an angular step size of 0.005°·s^-1^.

The textural properties were investigated by nitrogen physisorption measurements at −196 °C using a Quantachrome Autosorb iQ3 gas sorption analyser. Before analysis, SBA-15, OMC_FA_, and OMC_GLY_ were degassed at 150 °C for 30 min (5 °C·min^-1^), followed by a treatment at 300 °C for 90 min (5 °C·min^-1^). Regarding the gold carbon replica Au@OMC_GLY_, the pre-treatment was carried out at 100 °C for 30 min (5 °C·min^-1^), followed by a temperature increase to 150 °C for 90 min (5 °C⋅min^-1^). The specific surface area (sBET) and the pore volume were determined by applying the BET (Brunauer-Emmet-Teller) and BJH (Barrett-Joyner-Halenda) methods, respectively.

Scanning electron microscopy (SEM) was used to study the morphology of the synthesized materials, employing a PhenomProX coupled with Energy Dispersive X-ray (EDX) microanalysis for elemental investigation, operating at 6·10^−6^ torr and 15 kV.

Raman spectroscopy of carbon-based electrocatalysts was performed using a Labram HR 800 spectrometer coupled with a LT3-OM Helium Flow Cryostat, with a laser wavelength of 532 nm, a diffraction grating of 1800 gr⋅mm^-1^, and an exposure time of 10 s.

### 2.3 Gas Diffusion Electrodes

The working electrodes were prepared by depositing each carbon material on a gas diffusion layer (GDL, 29 BC Sigracet®) with a geometric area of 10 cm^2^ to obtain Gas Diffusion Electrodes (GDEs), acting as anodes. Precisely, each specific catalyst was mixed with an appropriate amount of ethanol (solvent) and Nafion solution (10% in ethanol) as a binder and then sonicated for 10 min. The resulting ink was deposited by means of an airbrush to obtain a homogeneous layer onto a GDL placed on a plate heated to about 100 °C to facilitate solvent evaporation. The final loading for each catalyst was about 1 mg⋅cm^-1^.

### 2.4 Electrochemical measurements

**FIGURE 1:**
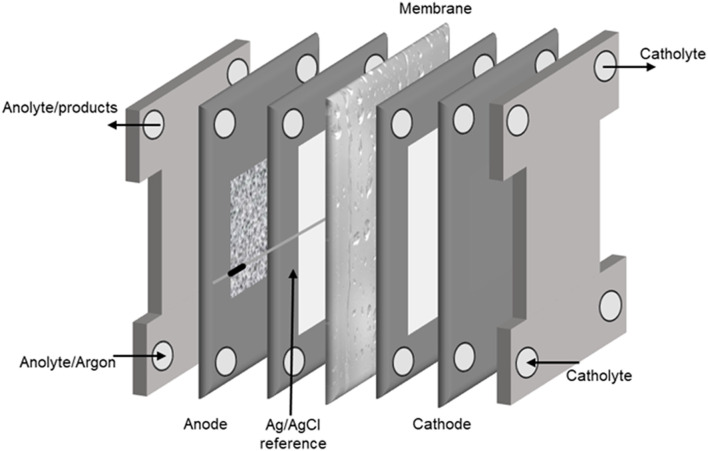
Scheme of the electrocatalytic microflow cell.

Furfural oxidation was carried out at ambient temperature and pressure in a three-electrode microflow cell (ElectroCell) using a potentiostat/galvanostat (Autolab PGSTAT302N, Metrohm). A 1 mm Ag/AgCl (3.5 M KCl) leak-free electrode and a Pt plate were employed as the reference and counter electrodes, respectively, while the working electrode was the GDE prepared following the methodology previously reported. Anodic and cathodic compartments were separated by a proton exchange membrane (Nafion 115). Before its use, the Nafion membrane was treated with 3 %wt H_2_O_2_ at 80 °C for 1 h to remove organic impurities and then activated by using a 0.5 M solution of H_2_SO_4_ at the same temperature for another hour, followed by washing with deionized H_2_O until pH = 7. A volume of 40 mL of anolyte and 40 mL of catholyte was used, with each solution circulated through the cell at a flow rate of 15 mL⋅min^-1^ using a peristaltic pump. Before each test, the anolyte solution was saturated with pure argon for 40 min to avoid possible interferences from oxygen, and a 10 mL⋅min^-1^ argon flow was maintained throughout the experiments. A schematic of the cell configuration is shown in Figure 1.

Chronoamperometric measurements were performed at different potentials in three electrolytes (1 M KOH, 0.5 M H_2_SO_4_, and 0.25 M HClO_4_) to investigate the effects of electrolyte nature on the furfural oxidation reaction. Before the electrocatalytic tests, cyclic voltammetry was performed in all the electrolyte solutions over the 0–1.8 V range (vs. Ag/AgCl) at a scan rate of 5 mV⋅s^-1^, both in the presence and absence of furfural, to obtain information on the electrochemical behaviour of the catalysts. All electrochemical measurements (including Faradaic efficiencies, conversion values, and Tafel slopes) were performed at least in duplicate to ensure statistical reliability and reproducibility. The reported data represent the average of independent experiments, and the error bars shown in the figures correspond to the standard deviation.

The products obtained in the liquid phase were analysed by a High Performance Liquid Chromatography (HPLC) system (Shimadzu Nexera-I LC-2040C) equipped with an Aminex HPX-87H column and a photo diode array (PDA) detector. In contrast, an online Agilent 490 micro gas chromatograph (Micro GS), equipped with Molsieve 5Å and PoraPlot Q columns, was used for the identification and quantification of gas-phase products.

Faradaic efficiency (FE), were calculated by comparing the amount of product formed during the experiments with the total charge passed during the electrolysis (1 h) using the following formula:

$$FE=\frac{n\cdot z\cdot F}{Q}\times 100$$

Where *n* are mols of formed products quantified by HPLC, *z* are electrons involved in the reaction (e.g., 6 for 5-HFN), *F* is the Faraday Constant (96485 C), *Q* is Total Charge (i⋅t).

## 3 Results and discussion

### 3.1 Structural and textural characterization

**FIGURE 2:**
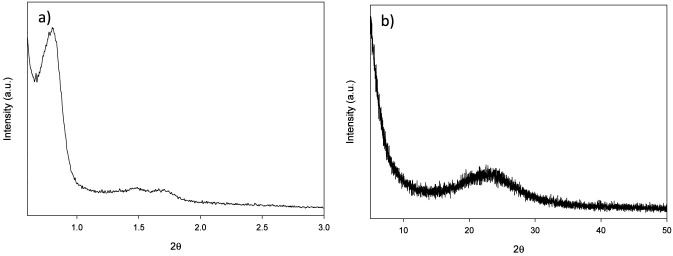
XRD pattern of SBA-15 registered at low **(a)** and high **(b)** angles, respectively.

The structure of SBA-15 was analysed using low- and high-angle XRD, with the corresponding diffraction patterns shown in Figures 2a,b. The low-angle diffraction pattern of SBA-15 exhibits three well-resolved peaks at 2θ = 0.8°, 1.5°, and 1.6°, indexed to the (100), (110), and (200) reflections, respectively, consistent with the hexagonal p6 mm symmetry typical of SBA-15. The broad reflection at 2θ ≈ 23° observed in the high-angle region is characteristic of amorphous silica, with its low intensity suggesting a relatively low degree of long-range order (Armandi et al., 2008; Reddy Police et al., 2015).

**FIGURE 3:**
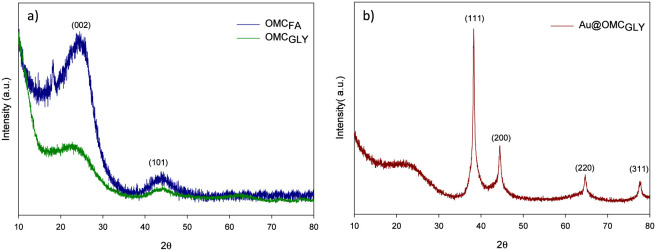
XRD pattern of OMCs materials **(a)** and Au@OMC_GLY_ **(b)**.

The XRD patterns of the carbon replicas OMC_FA_ and OMC_GLY_ are presented in Figure 3a. Both samples display a broad peak at 2θ ≈ 23°, corresponding to the (002) plane of graphitic carbon. OMC_GLY_ additionally exhibits a peak at 2θ ≈ 43°, attributable to the (101) plane, indicative of a more ordered structure (Ignat et al., 2010). The presence of this reflection, characteristic of both hexagonal and rhombohedral phases, confirms the formation of partially graphitized walls in the mesoporous carbon.

Following the deposition of gold nanoparticles (AuNPs) onto OMC_GLY_, the XRD pattern (Figure 3b) shows additional peaks at 2θ = 38°, 44°, 64°, and 78°, corresponding to the (111), (200), (220), and (311) planes of face-centered cubic (fcc) metallic gold, respectively (Papanikolaou et al., 2021). These reflections confirm the successful incorporation of crystalline AuNPs within the carbon matrix. The average crystallite size of AuNPs on OMC_GLY_, estimated via the Scherrer equation, was approximately 21 nm, indicating the formation of well-dispersed nanosized gold without significant aggregation, consistent with the preservation of the mesostructured order.

**FIGURE 4:**
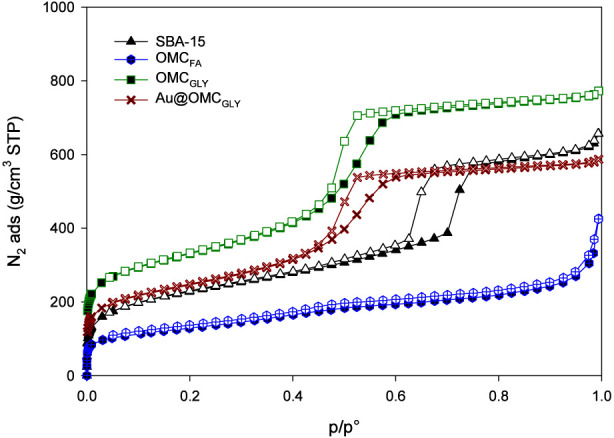
Nitrogen adsorption-desorption isotherms of SBA-15, OMC_FA_, OMC_GLY_ and Au@ OMC_GLY_ materials.

Nitrogen adsorption-desorption measurements were performed to assess the textural properties of all synthesized materials (Figure 4). SBA-15 exhibits a type IV isotherm with a type H1 hysteresis loop, characterized by a sharp adsorption step at p/p_0_ ≈ 0.7, indicative of uniform mesopores (Thommes et al., 2015). OMC_GLY_ and Au@OMC_GLY_ display similar adsorption behavior, confirming their mesoporous nature with predominantly cylindrical pores. In contrast, OMC_FA_ exhibits a combination of type II and IV features with H3 hysteresis, typical of macro-mesoporous materials.

Although carbon replicas generally exhibit higher surface areas than the silica template, in this case, OMC_FA_ shows a lower specific surface area (459 m^2^⋅g^-1^) than SBA-15 (811 m^2^⋅g^-1^). The decrease in surface area related to OMCFA could be ascribed to a lower order of the structure, as also confirmed by the XRD results. In fact, as reported by Janus et al. (2020), during the pore filling of the template, a random accumulation of the carbon precursor into the pores and, therefore, the furfuryl alcohol may have only partially occupied the pores of the SBA-15. Moreover, the possible formation of polyfurfuryl alcohol blocks at the entrance of the pores could decrease porosity, thereby reducing pore volume. In contrast, OMC_GLY_ demonstrates a higher surface area (1160 m^2^⋅g^-1^), suggesting the formation of micropores and additional external carbon. As regards Au@OMC_GLY_, the decrease in the surface area in comparison to OMC_GLY_ could be due to the inclusion of the gold nanoparticles into the pores of the carbon replica. Following the deposition of the AuNPs, it's possible to observe a decrease in the pore volume from 1.2 to 0.9 cm^3^⋅g^-1^ (Table 1).

**TABLE 1:** Textural properties of the SBA-15, carbon replicas, and gold nanoparticles supported on carbon replica.

Sample	S_T_^BET^ ^a^ (m^2^⋅g^-1^)	S_µ_ ^b^ (m^2^⋅g^-1^)	V_µ_ ^b^ (cm^3^⋅g^-1^)	V_m_ ^b^ (cm^3^⋅g^-1^)	V_p_ ^b^ (cm^3^⋅g^-1^)
SBA-15	811	222	0.10	1.4	1.5
OMC_FA_	459	55	0.02	0.6	0.6
OMC_GLY_	1160	281	0.13	1.1	1.2
Au@OMC_GLY_	857	116	0.06	0.8	0.9

^a^ S_T_^BET^: specific total surface area.

^b^ S_µ_, V_µ_, V_m_: specific microporous area, volume, and mesoporous volume; V_p_: total pore volume.

**FIGURE 5:**
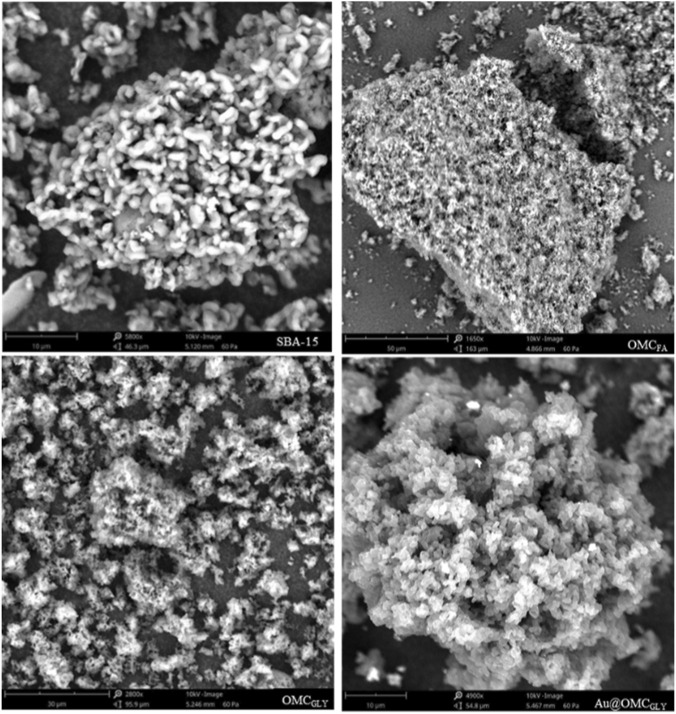
SEM images of SBA-15 and synthesized OMC_FA_, OMC_GLY_ and Au@OMC_GLY_.

SEM analysis (Figure 5) reveals that SBA-15 exhibits the typical elongated fibrous morphology (Abate et al., 2011; Gheno et al., 2016), partially retained in both carbon replicas. No significant morphological changes are observed after AuNP deposition, since the gold nanoparticles are scarcely visible at the 10 µm magnification used for imaging. However, the presence of AuNPs on carbon replicas was fully confirmed by EDX analysis, although the detected gold percentage (1%) was lower than the expected theoretical amount percentage (5%). Probably, some of the gold nanoparticles were incorporated into the channels of the carbon supports and were not detectable by EDX analysis. A further confirmation of this hypothesis is also provided by the results regarding the surface area of Au@OMC_GLY_, which shows a lower surface area than OMC_GLY_, as a direct consequence of the occlusion of the pores by the AuNPs.

**FIGURE 6:**
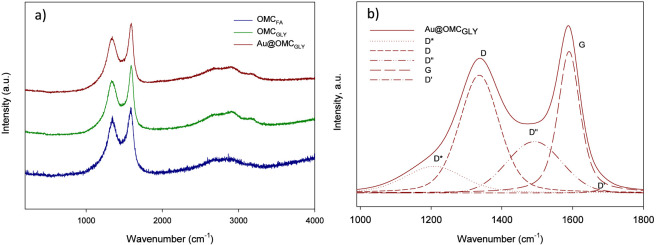
Raman spectra of pristine OMCs and Au@OMC_GLY_ **(a)**, and corresponding deconvoluted spectrum of Au@OMC_GLY_ **(b)**.

Raman spectroscopy was used to assess the degree of order of the carbon materials (Figure 6a). All carbon replicas display the characteristic D (∼1350 cm^-1^) and G (∼1590 cm^-1^) bands. The G band corresponds to the E2 g vibration mode of graphite of the sp2-bonded carbons, while the D band arises from defects and disorder (Ma et al., 2019). The presence of defects and consequently the disorder of the structure is determined through the calculation of the I_D_/I_G_ intensity ratio of the D and G bands. Moreover, to accurately account for other bands contributing to Raman scattering in the two carbon replicas, the spectra were fitted with a combination of four Lorentzian and one Gaussian functions (Jurkiewicz et al., 2018).

As a result of the fitting, we can distinguish five components contributing to the first-order Raman spectrum, which were assigned to D*, D, Dʺ, G, and Dʹ (Figure 6b). The D* band is located around 1220 cm^-1^, and its presence is associated with the vibration of sp^3^ carbons bonded to oxygen-containing groups (Lee et al., 2021; Papanikolaou et al., 2022b). Instead, Dʺ located at 1490 cm^-1^ is related to a decrease of the crystallinity and the Dʹ band at (∼1650 cm^−1^) related to “graphene-like” layers or sp^2^ nanostructures activated by defects (Li et al., 2023a; Muzyka et al., 2018). In Table 2, the I_D_/I_G_ intensity ratios obtained from Raman-spectrum fits are reported.

**TABLE 2:** I_D_/I_G_, I_D*_/I_G_ and I_D″_/I_G_ values calculated by five functions deconvolution.

Sample	I_D_/I_G_	I_D*_/I_G_	I_D″_/I_G_
OMC_FA_	1.26	0.42	0.68
OMC_GLY_	1.12	0.51	0.55
Au@OMC_GLY_	1.34	0.43	0.72

From the analysis of the intensity ratio, we observe that the carbon replica obtained using FA shows a slightly more disordered structure than that obtained using GLY.

Moreover, the intensity of the Dʹ band of OMC_GLY_ is higher than that of OMC_FA_, indicating a greater graphene-like character. Following the deposition of Au NPs, the order degree of OMC_GLY_ shows a different behaviour, leading to a more disordered structure while maintaining a more pronounced graphene character. Defect sites such as edge planes, vacancies, and sp^3^-hybridized domains can act as active centers in carbon electrocatalysts, by facilitating adsorption and activation of reactant molecules (Xue et al., 2020). An increased defect density is often associated with enhanced catalytic activity and improved reaction kinetics, typically reflected in lower Tafel slopes and reduced activation barriers in electrochemical processes (Xue et al., 2020; Zhang et al., 2020). However, catalytic efficiency does not depend solely on defect density. While structural disorder provides active sites, an adequate degree of graphitic ordering is essential to ensure efficient charge transport across the electrode (Pérez-Rodríguez et al., 2019). Therefore, optimal electrocatalytic performance generally arises from a balanced interplay between defect density and graphitic conductivity. In this context, OMC_GLY_, which exhibits a slightly lower I_D_/I_G_ ratio than OMC_FA_ but a more pronounced graphene-like character, may benefit from a more favorable compromise between accessible active sites and electronic conductivity.

Analysing the second-order Raman spectrum in the range from 2500 to 3200 cm^-1^ we observe three additional bands. The 2D band at 2700 cm^-1^ is the overtone of the D band generated by a double resonance-enhanced two-phonon process. In contrast to the D band, the 2D band does not require the presence of defects and is thus always visible in the second-order Raman spectra of sp2 carbon. The D + G band at 2940 cm^-1^ is the combined overtone of the D and G bands, and the 2G band at 3170 cm^-1^ is related to the overtone of the G band. The 2D, D + G, and 2G bands are highly structure-sensitive; in fact, an increase in defects in the carbon replica material affects inelastic phonon scattering, causing a decrease in the intensity of the 2D band (Bharath and Banat, 2021; Bokobza et al., 2015).

### 3.2 Linear sweep voltammetry

**FIGURE 7:**
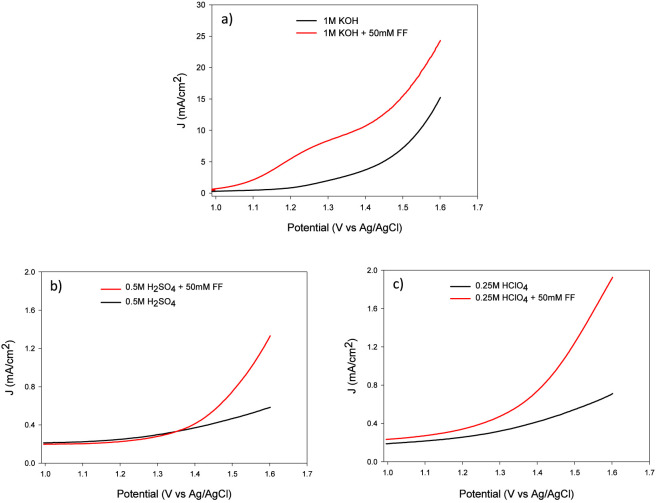
Linear sweep voltammograms of OMC_FA_ in 1 M KOH **(a)**, 0.5M H_2_SO_4_ **(b)**, and in 0.25M HClO_4_ **(c)** in the absence (dark line) and presence of 50 mM of furfural (red line).

The behaviour of the redox properties of all prepared electrodes was investigated by means of Linear sweep voltammetry (LSV) in three different electrolytes (1M KOH, 0.5M H_2_SO_4_ and 0.25M HClO_4_). Figure 7 shows the voltammograms measured in the different electrolyte solutions for the OMC_FA_ in the absence and presence of 50 mM of furfural, black and red lines, respectively. In the presence of only electrolyte, the inflection of the curve, due to the water oxidation reaction, takes place at higher values of potential. On the contrary, when furfural is present in the electrolyte solution, the inflection appears at lower potentials than in water oxidation. The sudden increase in current (1.1V–1.4 V vs. Ag/AgCl) on the anodic scan is due to the oxidation of furfural; at higher potentials (>1.5 V), the oxygen evolution reaction (OER) becomes predominant.

### 3.3 Electrocatalytic activity

The type and nature of the electrolyte can strongly affect the pathways of furfural conversion. In this regard, before electrocatalytic anodic oxidation of furfural, an initial study was conducted using the OMC_FA_ material in three electrolytes (KOH, H_2_SO_4_, and HClO_4_) to determine the optimal operating conditions. All preliminary chronoamperometric tests were carried out based on the LSV results.

**FIGURE 8:**
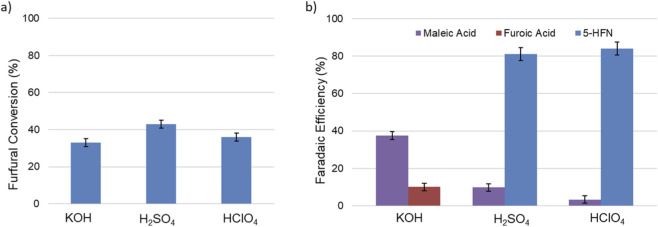
Furfural conversion in three different electrolytes **(a)** and Faradaic efficiency to products **(b)** by using the OMCFA.

Figure 8a shows the results obtained with the OMC_FA_ in the three different electrolytes after 1 h of reaction at 1.4 V (vs. Ag/AgCl). In an alkaline medium, the lowest furfural conversion was observed (33%), whereas an increase was observed when acidic electrolytes were employed (36% HClO_4_ and 43% H_2_SO_4_). Although the furfural conversion values were similar across all electrolytes, the product distribution was strongly affected. In fact, when the reaction is carried out in an acidic environment, the main product is 5-HFN, followed by the formation of small amounts of maleic acid. Instead, in the presence of a basic ionic medium, the conversion of furfural leads to the formation of furoic acid and maleic acid (Figure 8b). The formation of furoic acid in the presence of a basic electrolyte could be attributed to the Cannizzaro disproportionation reaction, a reaction in which aldehydes, without hydrogen atoms in α-position with respect to the aldehyde group, are involved (Douthwaite et al., 2017). This hypothesis was also confirmed by blank tests carried out in KOH containing 50 mM of furfural in the presence of the catalyst powder and in the absence of current, leading to the equimolar formation of furoic acid and furfuryl alcohol. This test confirmed that the amounts of furoic acid and furfuryl alcohol produced during the electrocatalytic tests were formed by the Cannizzaro reaction promoted by the catalyst in the heterogeneous phase. Similar blank tests were also carried out in the two acidic environments, where no oxidation products were observed, confirming that the products obtained in both acidic media derive exclusively from the electrocatalytic oxidation of furfural. Moreover, higher selectivity for 5-HFN formation was observed with HClO_4_, prompting us to select this electrolyte for the study of the electrocatalytic activity of all the synthesized electrocatalysts.

Based on the preliminary tests, the catalytic activity of OMC-based electrocatalysts was evaluated in 0.25M HClO_4_ by applying two different potentials, 1.4 V and 1.5 V (vs. Ag/AgCl), and the results of the electrocatalytic behaviour were compared with those obtained by employing a reduced graphene oxide (rGOx) based electrode used as a benchmark. All tests were carried out using a microflow cell in a three-electrode configuration for 1 h of reaction.

**SCHEME 1:**
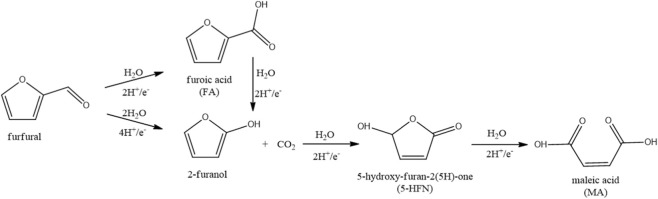
Possible furfural oxidation pathways.

The analysis of the liquid phase showed that all the electrocatalysts tested produced two main compounds: 5-HFN and maleic acid, with the latter present in smaller amounts. Instead, the gas-phase analysis revealed traces of H_2_, likely generated in the cathodic compartment via the water-splitting reaction and partially diffusing into the anodic compartment through the Nafion membrane. Moreover, CO_2_ was detected in the anodic feed, and its concentration increased with increasing potential. The CO_2_ production could be associated with two different pathways: i) the initial decarboxylation process of furfural to form 2-furanol, a reaction intermediate which subsequently leads to the formation of 5-HFN, or ii) the decarboxylation reaction of furoic acid, which also can be produced by the furfural oxidation (Scheme 1) (Román et al., 2019).

**FIGURE 9:**
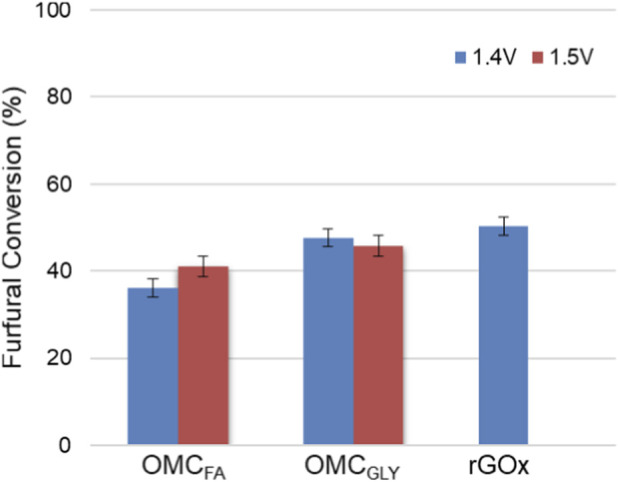
Furfural conversion in OMC-based materials and comparison with rGOx benchmark.

In Figure 9, the conversion of furfural for both OMC-based materials, compared to the rGOx benchmark, is reported after 1 h of reaction. As can be observed, all systems lead to a fairly similar furfural conversion, ranging from 36% to 50% and the difference between the two applied potentials concerning OMCs slightly affects the furfural conversion.

**FIGURE 10:**
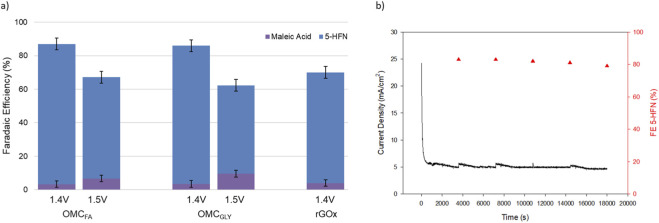
Faradaic efficiency of OMC-based materials and comparison with the rGOx benchmark **(a)** and 5-h stability test of OMG_GLY_ at 1.4 V **(b)**.

However, it is closely related to the electrochemical selectivity to the main products (5-HFN and maleic acid). As can be noticed (Figure 10a), FE towards 5-HFN reaches higher values at the lowest potential (∼80%). Conversely, increasing the applied potential reduced the FE for 5-HFN production, accompanied by a simultaneous increase in the FE for maleic acid formation. These results outperform the rGOx benchmark, which, at 1.4 V, achieved a FE of 66% to 5-HFN while maintaining a constant 4% for maleic acid. Considering the results obtained, OMC_GLY_ appears to be the best-performing electrocatalyst, with a furfural conversion of 48% and a FE of 83% to 5-HFN at 1.4 V. The stability of OMG_GLY_ was assessed on 5-h test without relevant loss in the catalytic activity reaching a final FE around 80% (Figure 10b).

The improved performances obtained by OMC_GLY_ could be attributed to its structure, which, as highlighted by BET analysis, exhibits a mesoporous component with a microporous contribution, resulting in an increased surface area and pore volume that may favour the interaction with the active species. Additionally, the presence of a more graphitic character, as indicated by the D band observed in Raman spectroscopy, could improve electrical conductivity, thereby enhancing electrocatalytic performance. Although it is well known that rGOx demonstrates high electrical conductivity, its lower electrocatalytic activity could be attributed to its reduced pore volume and less ordered structure (I_D_/I_G_ = 3.26) compared to the OMC_FA_ (I_D_/I_G_ = 1.26) and OMC_GLY_ (I_D_/I_G_ = 1.12), which is the material with the highest order degree.

To gain deeper insight into surface reactivity, AuNPs were integrated onto OMC_GLY_ to investigate how localized electronic enrichment and metal–support interactions influence the oxidation pathway (Parmentier et al., 2004; Liu et al., 2017).

**FIGURE 11:**
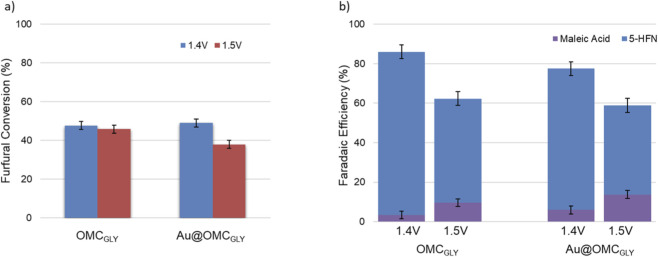
Furfural conversion **(a)** and Faradaic efficiency **(b)** of OMC_GLY_ and Au@OMC_GLY_ electrodes.

In Figure 11a, the furfural conversion of Au@OMC_GLY_ is reported in comparison to the corresponding metal-free OMC_GLY_. No notable differences are observed at 1.4 V (vs. Ag/AgCl), with the conversion about 49%. On increasing the applied potential (1.5 V), a decrease in furfural conversion is recorded (38%).

**FIGURE 12:**
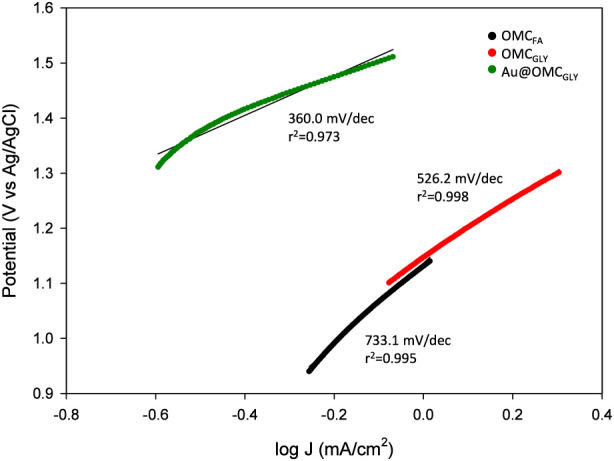
Tafel plots of electrocatalysts for the anodic furfural oxidation.

Although the introduction of AuNPs appears to have no significant impact on the electrocatalytic behavior of OMC_GLY_ regarding furfural conversion, the analysis of electrocatalytic performance reveals a notable difference in terms of selectivity towards primary reaction products. As illustrated in Figure 11b, at both potentials, the FE to 5-HFN decreases, favoring the formation of maleic acid, which reaches its highest FE of 14% at 1.5 V. A possible explanation for these results is that the presence of metal nanoparticles may enhance the electron transfer. This would favor the opening of the furan ring of 5-HFN and, consequently, increase selectivity towards the formation of maleic acid. Further evidence is provided by the Tafel slope analysis shown in Figure 12. Lower Tafel slopes typically indicate a faster electrocatalytic reaction. Notably, Au@OMC_GLY_ exhibits the lowest Tafel slope of 360 mV·dec^−1^, followed by OMC_GLY_ and OMC_FA_, with values of 526.2 mV·dec^−1^ and 733.1 mV·dec^−1^, respectively. In this context, the observed trend can be rationalized by considering also the structural characteristics derived from Raman analysis. Although OMC_GLY_ exhibits a slightly lower I_D_/I_G_ ratio than OMC_FA_, indicating a more ordered graphitic framework, the balance between defect density and graphitic conductivity appears to govern the reaction kinetics. While defects provide active sites, an adequate degree of graphitic ordering ensures efficient electron transport, suggesting that optimal catalytic performance arises from a synergistic interplay between structural disorder and electronic conductivity rather than from maximum defect density alone (Pérez-Rodríguez et al., 2019).

We can propose a possible reaction pathway for furfural oxidation based on the experimental results and literature data (Parthasarathy et al., 2006; Ignat et al., 2010). As illustrated in Scheme 1, furfural oxidation can proceed through different reaction pathways, leading either to furoic acid or to 2-furanol as primary intermediates.

However, we are inclined to exclude the pathway involving furoic acid as an intermediate since its direct electrocatalytic oxidation, under identical conditions, resulted in negligible formation of both 5-HFN (FE < 11%) and maleic acid (FE < 3%) with poor furoic acid conversion (< 13%). This finding suggests that the production of 5-HFN does not proceed primary via furoic acid as intermediate, since its direct oxidation did not produce significant amounts of these products. Moreover, furoic acid was never detected during the electrocatalytic oxidation of furfural with all the developed OMC electrocatalysts. As shown in Figures 10a, 11b, 5-HFN and maleic acid were the only products observed.

Although multiple mechanistic pathways are theoretically possible, our experimental evidences strongly suggest that the most plausible conversion route of furfural to 5-HFN proceeds via 2-furanol, which subsequently evolves into 5-HFN, responsible for the formation of maleic acid (Hasse et al., 2023; Li et al., 2023b). Furthermore, no additional oxidation products were detected throughout the experiments, indicating that maleic acid does not undergo further transformation under the investigated reaction conditions.

## 4 Conclusions

This work describes the successful synthesis, using furfuryl alcohol and glycerol as carbon precursors, of ordered mesoporous carbon materials from SBA-15 silica templates and their further characterization as electrocatalysts in the mild oxidation of furfural. Also presented is a detailed structural and textural analysis, characterizing highly ordered mesoporous frameworks with tunable surface area, pore volume, and graphitic character. The OMC derived from glycerol presents the highest surface area, along with a micro-mesoporous structure in which the ordering is more pronounced, which is in line with the superior electrocatalytic performance. Raman spectroscopy indicated lower defect density and enhanced graphitic domains in this sample, a feature that may contribute to improved charge transfer and electrochemical stability.

Electrochemical studies revealed that the electrolyte environment strongly affected the furfural oxidation pathway. Acidic media, in particular, 0.25 M HClO_4_, favored higher selectivity toward 5-HFN, while alkaline conditions favored furoic acid formation by a non-electrochemical Cannizzaro pathway. Among the three catalysts studied, OMC_GLY_ achieved the highest Faradaic efficiency for 5-HFN of 83% at 1.4 V vs. Ag/AgCl, surpassing that of OMC_FA_ and even the benchmark rGOx electrode. Depositing AuNPs onto OMC_GLY_ (Au@OMC_GLY_) did not improve furfural conversion but significantly altered product selectivity, increasing the formation of maleic acid, an effect which was interpreted by Tafel plot analysis as facilitation of electron transfer and probable furan-ring opening on Au sites.

The combined analysis of electrochemical performance and structural characterization clearly demonstrates that textural order, electronic conductivity, and pore accessibility play a decisive role in governing both activity and selectivity in carbon-based electrocatalysts. In contrast to previously reported metal-based systems for furfural electro-oxidation, the present study shows that a rationally engineered, metal-free ordered mesoporous carbon can achieve a high FE toward 5-HFN (up to 83%) under acidic conditions in a continuous microflow cell. The advancement is therefore not limited to improved selectivity but also lies in the establishment of a clear structure-activity-selectivity relationship, linking graphitic character, degree of order, and hierarchical porosity to catalytic behavior.

The superior performance of OMC_GLY_ highlights the potential of tailored mesostructured carbons as efficient and scalable metal-free system for the selective electro-oxidation of biomass-derived molecules. Further studies will focus on optimizing surface functionalities and exploring strategies for co-doping or defect engineering to improve selectivity and durability in continuous-flow electrochemical systems.

## Data Availability

The raw data supporting the conclusions of this article will be made available by the authors, without undue reservation.
